# A100 HEREDITARY HEMORRHAGIC TELANGIECTASIA: MORE THAN JUST ANOTHER RECTAL BLEED

**DOI:** 10.1093/jcag/gwad061.100

**Published:** 2024-02-14

**Authors:** D Leung, J Y Guo, S Zepeda-Gomez, S Wesilenko, B Halloran, D Vethanayagam

**Affiliations:** University of Alberta Faculty of Medicine & Dentistry, Edmonton, AB, Canada; University of Alberta Faculty of Medicine & Dentistry, Edmonton, AB, Canada; University of Alberta Faculty of Medicine & Dentistry, Edmonton, AB, Canada; University of Alberta Faculty of Medicine & Dentistry, Edmonton, AB, Canada; University of Alberta Faculty of Medicine & Dentistry, Edmonton, AB, Canada; University of Alberta Faculty of Medicine & Dentistry, Edmonton, AB, Canada

## Abstract

**Background:**

Hereditary hemorrhagic telangiectasia (HHT), previously known as Osler-Weber-Rendu syndrome, is an autosomal dominant disorder characterized by widespread telangiectasia and visceral arteriovenous (AV) malformations. Diagnosis of HHT is commonly guided by The Curaçao Diagnostic Criteria. Molecular genetics can help confirm the clinical diagnosis. Genes involved include ENG, ACVRL1, SMAD4, and GDF2, which affect the bone morphogenic protein signaling pathway important in maintaining vascular endothelial integrity.

**Aims:**

This report aims to review the case of a young male from a rural community in Northern Alberta with a possible new diagnosis of HHT, presenting with rectal bleeding in the setting of family history of telangiectasias.

**Methods:**

Retrospective review of one patient.

**Results:**

We report the case of a 33-year-old male who presented with a 1-day history of rectal bleeding (bright red turning to black) with hemoglobin at 116g/L. Past medical history was significant for occasional epistaxis since age 8, previous gastrointestinal bleed and laparotomy with pathology showing small intestinal vascular malformation at the age of 15, and small bowel obstruction secondary to adhesions requiring resection at the age of 26. There was no history of iron deficiency anemia. Family history was positive for a mother who had Von Willebrand disease and partial colectomy at the age of 28 due to bowel telangiectasias. His mother had no formal HHT diagnosis, but it was unclear if molecular genetics had been performed. Inpatient colonoscopy showed no culprit lesions but free-floating blood clots throughout the colon (Image). EGD and push enteroscopy were normal. CT enterography was then performed showing AV fistula in the distal ileum and venous malformations in the jejunum. Video capsule endoscopy followed revealed a few non-bleeding telangiectasia and petechiae. The patient was assessed to have possible HHT by The Curaçao Diagnostic Criteria. He was supplemented with IV iron and started on short-acting octreotide. On discharge, he received funding approval for long-acting octreotide and was referred to the Edmonton HHT clinic for follow up and further screening.

**Conclusions:**

HHT remains underdiagnosed in both children and adults. Maintaining a strong suspicion and prompt diagnosis via the Curaçao Criteria is crucial for appropriate screening of the multisystem complications which stem from the AV malformations. This multidisciplinary approach can aid in reducing morbidity and mortality.

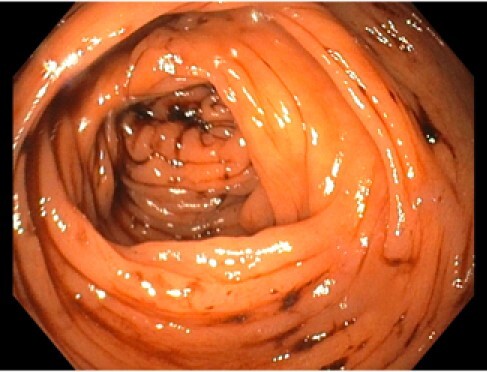

**Funding Agencies:**

None

